# Alterations in purine and pyrimidine metabolism associated with latent tuberculosis infection: insights from gut microbiome and metabolomics analyses

**DOI:** 10.1128/msystems.00812-24

**Published:** 2024-10-22

**Authors:** Boyi Yang, Xiaojing Guo, Chongyu Shi, Gang Liu, Xiaoling Qin, Shiyi Chen, Li Gan, Dongxu Liang, Kai Shao, Ruolan Xu, Jieqing Zhong, Yujie Mo, Hai Li, Dan Luo

**Affiliations:** 1 Department of Biostatistics, School of Public Health and Management, Guangxi University of Chinese Medicine, Nanning, China; 2 Department of Physiology, School of Basic Medical Sciences, Guangxi Medical University, Nanning, China; 3 The First Clinical College, Guangxi Medical University, Nanning, China; 4 Molecular Biology Laboratory of Respiratory Disease, College of Laboratory Medicine, Chongqing Medical University, Chongqing, China; 5 Guangxi Key Laboratory of Translational Medicine for Treating High-Incidence Infectious Diseases with Integrative Medicine, Nanning, China; Zhejiang University College of Animal Sciences, HangZhou, Zhejiang, China

**Keywords:** latent tuberculosis infection, active tuberculosis, gut microbiome, metabolomics, purine metabolism, pyrimidine metabolism

## Abstract

Individuals with latent tuberculosis infection (LTBI) account for almost 30% of the population worldwide and have the potential to develop active tuberculosis (ATB). Despite this, the current understanding of the pathogenesis of LTBI is limited. The gut microbiome can be altered in tuberculosis patients, and an understanding of the changes associated with the progression from good health to LTBI to ATB can provide novel perspectives for understanding the pathogenesis of LTBI by identifying microbial and molecular biomarkers associated therewith. In this study, fecal samples from healthy controls (HC), individuals with LTBI and ATB patients were collected for gut microbiome and metabolomics analyses. Compared to HC and LTBI subjects, participants with ATB showed a significant decrease in gut bacterial α-diversity. Additionally, there were significant differences in gut microbial communities and metabolism among the HC, LTBI, and ATB groups. PICRUSt2 analysis revealed that microbiota metabolic pathways involving the degradation of purine and pyrimidine metabolites were upregulated in LTBI and ATB individuals relative to HCs. Metabolomic profiling similarly revealed that purine and pyrimidine metabolite levels were decreased in LTBI and ATB samples relative to those from HCs. Further correlation analyses revealed that the levels of purine and pyrimidine metabolites were negatively correlated with those of gut microbial genera represented by *Ruminococcus_gnavus_group* (*R. gnavus*), and the levels of *R. gnavus* were also positively correlated with adenosine nucleotide degradation II, which is a purine degradation pathway. Moreover, a combined signature including hypoxanthine and xanthine was found to effectively distinguish between LTBI and HC samples (area under the curve [AUC] of training set = 0.796; AUC of testing set = 0.924). Therefore, through gut microbiome and metabolomic analyses, these findings provide valuable clues regarding how alterations in gut purine and pyrimidine metabolism are linked to the pathogenesis of LTBI.

**IMPORTANCE:**

This study provides valuable insight into alterations in the gut microbiome and metabolomic profiles in a cohort of adults with LTBI and ATB. Perturbed gut purine and pyrimidine metabolism in LTBI was associated with the compositional alterations of gut microbiota, which may be an impetus for developing novel diagnostic strategies and interventions targeting LTBI.

## INTRODUCTION

Tuberculosis (TB), an ancient infectious disease caused by *Mycobacterium tuberculosis* (*M. tb*), remains a threat to global public health, especially in developing countries (1). Latent tuberculosis infection (LTBI) is a state of persistent immunity against *M. tb* without typical symptoms of active tuberculosis (ATB) during which *M. tb* is thought to be constrained by host immune responses (2, 3). Approximately 30% of the global population is estimated to have LTBI, of whom 5%–10% may develop ATB within the first 2 years after pathogen exposure (4–6), posing a major challenge to the control of the TB epidemic. Thus, the identification and prompt treatment of LTBI are effective approaches to reducing the transmission of ATB and the associated mortality rate (7). However, the current understanding of the pathogenesis of LTBI is limited (8).

Existing evidence indicates that gut microbiota and associated metabolites can regulate lung immunity, playing an important role in inflammatory and defensive responses (9). For example, gut microbes can produce short-chain fatty acids (SCFAs), which are circulated to the lung and bind to free fatty acid receptors expressed by alveolar macrophages and type II cells, thus promoting basal IL-1β expression and regulating the type 1 interferon (IFN) response to respiratory syncytial virus infection (10). Dysbiosis has been observed in many respiratory diseases, such as chronic obstructive pulmonary disease, asthma, SARS-CoV-2 infection, and TB (11–13). These findings suggest that characterizing the gut microbiota and associated metabolites may provide new insights for understanding the pathogenesis of LTBI. Gut microflora and their metabolites have shown potential for clinical application. Supplementation with probiotics, molecular preparation, and fecal microbiota transplantation are all approaches for the external modulation of the gut microbiota that have been tentatively employed for the treatment of non-digestive diseases such as depression (14), achieving positive effects. These advances suggest that manipulating the gut microbiota and metabolites associated therewith may be a viable therapeutic approach to managing LTBI, making the investigation of the gut microbial and metabolic profiles linked with this disease meaningful. Although previous studies have reported compositional alterations in gut microbiota, such as *Firmicutes*, *Bacteroidetes*, *Proteobacteria*, and *Actinobacteria*, in patients with ATB (15, 16), a few studies have investigated the gut microbiota in individuals with LTBI. In addition, the metabolic alterations in the gut microbiota caused by compositional dysbiosis remain poorly defined in patients with TB.

In this study, we sought to explore the gut microbial and metabolic differences among patients with ATB, LTBI, and healthy controls (HCs) using amplicon sequencing and untargeted metabolomics approaches. Through appropriate statistical analyses, we ultimately detected early functional alterations in the gut microbiota that arise after *M. tb* infection and metabolic features that can effectively discriminate between individuals with LTBI and HCs.

## MATERIALS AND METHODS

### Study design

**Fig 1:**
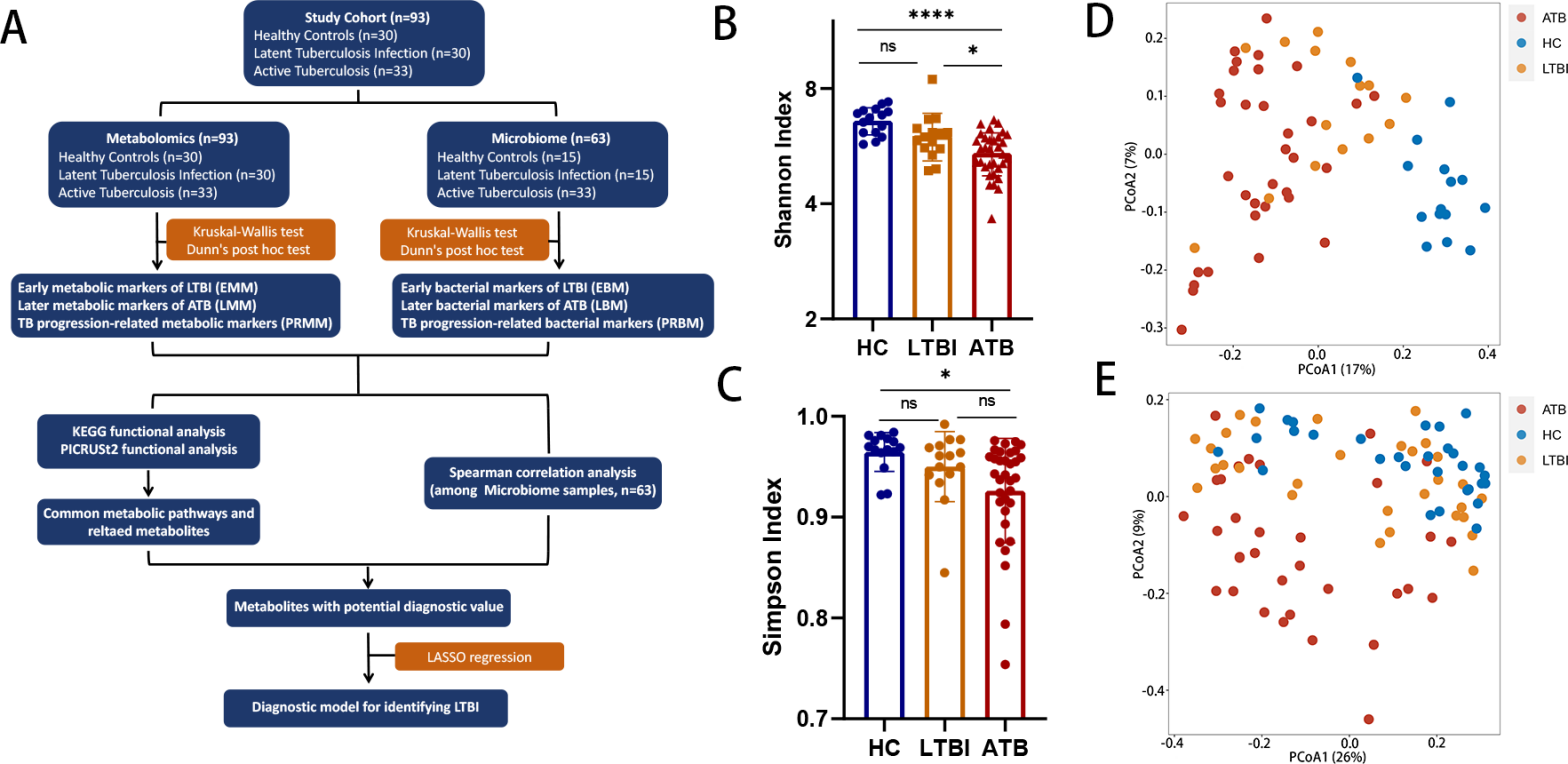
Tuberculosis is associated with broad changes in microbiome and metabolome profiles. (**A**) Flowchart presenting the design and analysis process of this study. This study adopts a case-control study design, with newly diagnosed active pulmonary tuberculosis patients as the ATB group, individuals with latent tuberculosis infections as the LTBI group, and healthy controls as the HC group. Fecal samples from the three groups were collected for 16S rRNA sequencing and untargeted metabolomics analyses to obtain the microbial features and metabolic profiling. Then, the differential microbial genera and metabolites were identified between each pair of groups, and correlation analyses were performed between metabolites and microbial genera to explore the molecular features associated with the onset and progression of LTBI. (**B, C**) ANOVAs revealing differences in the (**B**) Shannon and (**C**) Simpson index of fecal samples from ATB patients and HCs, with no significant differences between the HC and LTBI groups (^ns^*P >* 0.05, *P < 0.05, ****P < 0.0001). (**D, E**) Principal coordinate analysis (PCoA) of individuals undergoing (**D**) gut microbiome profiling and (**E**) gut metabolomic profiling, with plots based on the UniFrac distances. Each point represents a sample, and the colors represent different groups. The results of the PERMANOVA test to compare dissimilarity indexes among samples are shown above the plots.

The design and execution of the present study are summarized and simplified in a flowchart (Fig. 1A). We conducted this study in Pingnan County (TB case notification rate of 58.48 cases per 100,000 population per year) in the Guangxi Zhuang Autonomous Region of China. This study included 33 incident cases of ATB, 30 individuals with LTBI, and 30 HCs from Pingnan People’s Hospital, the exclusive designated medical institution for TB prevention and treatment in Pingnan. Patients with ATB and LTBI were diagnosed in accordance with the Technical Guidelines for Tuberculosis Prevention and Control in China issued by the Chinese Center for Disease Control and Prevention in 2021. Patients with ATB were recruited from the Department of TB Prevention and Treatment and were defined as follows: (i) clinical symptoms and chest imaging evidence; (ii) conclusive evidence of *M. tb* from at least one method including culture, GeneXpert MTB/RIF, and/or next-generation metagenome sequencing (mNGS) of sputum or bronchoalveolar lavage fluid samples; and (iii) no evidence of extrapulmonary TB. Individuals with LTBI and HCs were recruited from the physical examination center of Pingnan People’s Hospital. LTBI was defined by positive results from both the tuberculin skin test (0.1 mL dose of purified protein derivative, Chengdu, China; >10 mm) and QuantiFERON TB gold in-tube assay (Cellestis, Australia; >0.35 IU/mL) without any corresponding clinical symptoms and radiological features of ATB. HCs met the following inclusion criteria: (i) no clinical TB symptoms and chest imaging evidence; (ii) negative QuantiFERON TB gold in-tube assay; and (iii) no history of contact with patients with TB. The exclusion criteria for all three groups were as follows: (i) history of TB; (ii) anti-TB treatment before enrollment; (iii) simultaneously suffering from immunodeficiency or other comorbidities; (iv) <18 years of age; (v) samples were not collected in the morning; (vi) antibiotic or probiotic product use within the past 2 months; and (vii) history of gastrointestinal surgery. Detailed characteristics are presented in Table 1.

**TABLE 1:** Detailed characteristics of individuals enrolled in this study^*a*^

	Healthy controls	Latent tuberculosis infection	Active tuberculosis	*P*
Samples of metabolomics				
Total number	30	30	33	N/A^*b*^
Male, *n* (%)	15 (50.0)	13 (43.3)	13 (39.4)	0.695
Age (years), mean (SD)	49.1 (13.8)	45.7 (14.0)	48.3 (16.9)	0.657
BMI (kg/m^2^), mean (SD)	22.0 (2.2)	22.2 (2.4)	21.4 (2.5)	0.408
Samples of microbiome				
Total number	15	15	33	N/A
Male, *n* (%)	7 (46.7)	7 (46.7)	13 (39.4)	0.844
Age (years), mean (SD)	48.7 (13.2)	44.2 (15.8)	48.3 (16.9)	0.664
BMI (kg/m^2^), mean (SD)	21.5 (2.5)	22.2 (2.4)	21.4 (2.5)	0.625
Inclusion criteria				
Clinical symptoms	−	−	+	N/A
Abnormality in chest imaging	−	−	+	N/A
Tuberculin skin test	−	+	+	N/A
QuantiFERON TB gold in-tube assay	−	+	+	N/A
Positive culture for *M. tb*	Not conducted	Not conducted	12	N/A
Positive GeneXpert MTB/RIF	Not conducted	Not conducted	10	N/A
Postive *M. tb* identification by mNGS	Not conducted	Not conducted	29	N/A

^*a*^ Exclusion criteria of all the three groups: (i) history of TB, (ii) anti-TB treatment before enrollment, (iii) simultaneously suffering from immunodeficiency or comorbidities, (iv) <18 years, (v) samples were not collected in the morning, (vi) taken antibiotics or probiotic products within the past 2 months, and (vii) ever having undergone gastrointestinal surgery.

^*b*^ "N/A" indicates hypothesis testing was not conducted; "+," positive; "−," negative.

Samples from all of these 93 subjects were subjected to metabolic detection through an untargeted metabolomics approach (HC: *n* = 30; LTBI: *n* = 30; ATB: *n* = 33). However, of these 93 samples, only 63 samples underwent 16S rRNA gene amplicon sequencing (HC: *n* = 15; LTBI: *n* = 15; ATB: *n* = 33), with the 15 samples from the HC and LTBI groups having been randomly selected using random number generators in SPSS version 23.

### 16S rRNA gene sequencing

The total genomic DNA in fecal samples was extracted using the CTAB method, and the DNA concentration and purity were monitored using 1% agarose gels. DNA was diluted to 1 ng/µL with sterile water. Next, the V3–V4 regions of the 16S rRNA gene were amplified by PCR using primers (341F/806R) with barcodes. All PCRs contained 15 µL of Phusion High-Fidelity PCR Master Mix (New England Biolabs), 0.2 µM of each primer, and 10 ng of the target DNA, and the cycling conditions consisted of a first denaturation step at 98°C for 1 min, followed by 30 cycles of 98°C (10 s), 50°C (30 s), and 72°C (30 s), and a final 5 min extension at 72°C.

PCR products were mixed with an equal volume of 1× loading buffer (containing SYBR green), and then electrophoresis was performed on 2% agarose gels for DNA detection. The PCR products were mixed in equal proportions, and then a Qiagen Gel Extraction Kit (Qiagen, Germany) was used to purify the mixed PCR products.

Sequencing libraries were generated with NEBNext Ultra IIDNA Library Prep Kit (Cat No. E7645). The library quality was evaluated on the Qubit@ 2.0 Fluorometer (Thermo Scientific) and Agilent Bioanalyzer 2100 system. Finally, the library was sequenced on an Illumina NovaSeq platform and 250 bp paired-end reads were generated.

Raw tags were produced by merging the paired-end reads using FLASH (Version 1.2.11), and then, high-quality clean tags were obtained using fastp (Version 0.20.0) (17). The clean tags were compared to the Silva database using Vsearch (Version 2.15.0) to detect chimeric sequences, which were then removed to obtain the effective tags (18). Denoising of the effective tags was performed using QIIME2 (Version QIIME2-202006) to obtain initial amplicon sequence variants (ASVs), and then ASVs with a level <5 were filtered out. The absolute level of ASVs was normalized using a standard sequence number corresponding to the sample with the least number of sequences. Species annotation was also conducted using QIIME2 via the Silva Database.

### Bioinformatics analyses of the microbiome

Alpha diversity metrics, including the Shannon and Simpson indices, were calculated by QIIME2 and compared between groups using analyses of variances (ANOVAs) with Bonferroni *post hoc* multiple comparisons testing in SPSS version 23. For beta diversity analysis, principal coordinate analyses (PCoA) based on UniFrac distances were used to analyze the microbial community structures, and permutational multivariate analyses of variance (PERMANOVAs) were used to determine taxonomic differences between groups. Metabolic pathway analyses of the gut microbiota were performed using the MetaCyc database with PICRUSt2, and pathways that were different in level between the three groups were obtained using Kruskal–Wallis tests and Dunn’s post hoc test using the R packages “rstatix,” “dplyr,” “FSA,” and “rcompanion.” For differential analyses, microbial features annotated to a genus with an absolute level higher than 0 in at least 70% of the individuals were considered. The Kruskal–Wallis and Dunn’s *post hoc* tests performed in R software were applied to identify differential genera between the three groups, and *P*-values were subjected to Benjamini–Hochberg correction with a false discovery rate (FDR) threshold of 0.05.

### Untargeted metabolomics

Liquid chromatography-tandem mass spectrometry (LC-MS/MS) was used to detect the metabolites in fecal samples. The samples were individually ground with liquid nitrogen, and the homogenate was resuspended with prechilled 80% methanol via thorough vortexing. The samples were incubated on ice for 5 min and then centrifuged at 15,000 × *g* at 4°C for 20 min. Some of the supernatant was diluted to a final concentration of 53% methanol using LC-MS grade water. The samples were subsequently transferred to a fresh Eppendorf tube and then centrifuged at 15,000 *× g* at 4°C for 20 min. Finally, the supernatant was injected into the LC-MS/MS system for analysis (19).

UHPLC-MS/MS analyses were performed using a Vanquish UHPLC system (Thermo Fisher, Germany) coupled with an Orbitrap Q Exactive HF-X mass spectrometer (Thermo Fisher, Germany) at Novogene Co., Ltd. (Beijing, China). Samples were injected onto a Hypesil Gold column (100 mm × 2.1 mm, 1.9 µm) using a 12 min linear gradient at a flow rate of 0.2 mL/min. The eluents for the positive polarity mode were eluent A (0.1% formic acid in water) and eluent B (methanol). The eluents for the negative polarity mode were eluent A (5 mM ammonium acetate, pH 9.0) and eluent B (methanol). The solvent gradient settings were as follows: 2% B, 1.5 min; 2%–85% B, 3 min; 85%–100% B, 10 min; 100%–2% B, 10.1 min; and 2% B, 12 min. The Q Exactive HF-X mass spectrometer was operated in positive/negative polarity mode with a spray voltage of 3.5 kV, capillary temperature of 320°C, a sheath gas flow rate of 35 psi, an auxiliary gas flow rate of 10 L/min, a S-lens RF level of 60, and an auxiliary gas heater temperature of 350°C.

The raw data files generated by UHPLC-MS/MS were processed using Compound Discoverer 3.1 (CD3.1, Thermo Fisher) to perform peak alignment, peak picking, and quantitation for each metabolite. The main parameters were as follows: retention time tolerance, 0.2 min; actual mass tolerance, 5 ppm; signal intensity tolerance, 30%; signal/noise ratio, 3; and minimum intensity. Then, the peak intensities were normalized to the total spectral intensity, which was used to predict the molecular formula based on additive ions, molecular ion peaks, and fragment ions. The peaks were then matched with the mzCloud (https://www.mzcloud.org/), mzVault, and MassList databases to obtain accurate qualitative and relative quantitative results.

### Bioinformatics analyses of metabolomics data

PCoA was applied to visualize the metabolic differences between groups, similar to the approach employed for microbiome analyses. The variable importance in projections (VIP) value of each metabolite was calculated using the PLS-DA model. The selection of differential metabolites was conducted for the metabolites that were annotated in at least one platform (HMDB, KEGG, and Lipidmaps). The metabolite levels in the HC, LTBI, and ATB groups were compared using the Kruskal–Wallis test and Dunn’s *post hoc* test in R software, and *P*-values were subjected to Benjamini–Hochberg correction with an FDR threshold of 0.05. Then, differential metabolites were selected after screening using the cutoff values of “*P*_adj_ < 0.05,” “VIP > 1” (20, 21), and “fold change (FC) ≥ 1.5 or ≤ 0.66” (22, 23). To investigate metabolic pathways, Kyoto Encyclopedia of Genes and Genomes (KEGG) analysis was performed using MetaboAnalyst (Version 5.0).

### Correlation analyses

Spearman’s correlation analyses were conducted among the samples with both microbial and metabolic data (HC group: *n* = 15; LTBI group: *n* = 15; ATB group: *n* = 33) to evaluate the relationships between the gut microbiota and metabolites.

### Construction of a LASSO regression model

Least absolute shrinkage and selection operator (LASSO) regression was completed using the “glmnet” R package. All samples with metabolomics data from the HC group (*n* = 30) and LTBI group (*n* = 30) were divided into two subgroups. The samples with both microbiome data and metabolomics data were categorized as the training set (HC group: *n* = 15, LTBI group: *n* = 15), and the others, with only metabolomics data, were classified as the testing set (HC group: *n* = 15, LTBI group: *n* = 15).

### Statistical analyses

Statistical analyses were performed using SPSS version 23 (IBM, Armonk, NY, USA). Continuous data are expressed as the mean ± standard error of the mean and were compared using ANOVAs or Kruskal–Wallis tests based on the normality of the data distribution. Categorical data are expressed as percentages and were compared using the chi-squared test. Statistical significance was defined by a *P*-value < 0.05. Images were drawn using GraphPad Prism 9.0 and R software (Version 4.2.2).

## RESULTS

### TB infection induces shifts in gut microbiota composition and metabolism

Compared with the HC group, the ATB group exhibited significant decreases in the Shannon and Simpson indices (Fig. 1B and C; Shannon index: *P* = 5.688 × 10^−6^; Simpson index: *P* = 0.015). However, no significant differences in Shannon or Simpson indices were observed between the HC and LTBI groups. PCoA revealed that the HC group was largely separated from the LTBI and ATB groups (Fig. 1D; PERMANOVA: *R*^2^ = 0.164, *P* < 0.001). Interestingly, at the phylum level, 10 bacterial phyla, namely, *Acidobacteriota*, *Armatimonadota*, *Chloroflexi*, *Desulfobacterota*, *Gemmatimonadota*, *MBNT15*, *Myxococcota*, *Nitrospirota*, *Planctomycetota*, and *Verrucomicrobiota*, were clustered together, and their relative levels had apparently decreased to almost zero in the LTBI stage of TB as compared to HCs (Fig. S1A). At the class level, 18 clustered bacterial classes, including *Acidimicrobiia*, *Anaerolineae*, *Desulfobacteria*, *Desulfobulbia*, *Desulfuromonadia*, *Gemmatimonadetes*, *Holophagae*, *Ignavibacteria*, *Latescibacterota*, *MBNT15*, *PAUC43f_marine_benthic_group*, *Polyangia*, *Subgroup_22*, *Thermoanaerobaculia*, *Thermodesulfovibrionia*, *Vicinamibacteria*, *BD2-11_terrestrial_group*, and *NB1-j* exhibited the same trend (Fig. S1B). These findings highlight significant shifts in fecal microbiota composition in the early stage of TB infection. PCoA plots generated for metabolomic profiling showed that the ATB group was largely separated from the HC and LTBI groups (Fig. 1E; PERMANOVA: *R*^2^ = 0.103, *P* < 0.001).

### Differential analysis of the gut microbiota and associated metabolites

After Kruskal–Wallis/Dunn’s testing with Benjamini–Hochberg correction, 114 differential metabolites were identified between the LTBI and HC groups, 253 between the ATB and LTBI groups, and 344 between the ATB and HC groups. Based on trends in the variations of levels of these metabolites, three altered metabolite patterns were observed and summarized as early metabolic markers of LTBI (EMMs), later metabolic markers of ATB (LMMs), and TB progression-related metabolic markers (PRMMs) (Table S1). A total of 56 out of 67 metabolites from area 3 (Fig. S2A) were more or less abundant in the LTBI group relative to HCs, whereas they showed no significant alterations in terms of abundance between the LTBI and ATB groups (LTBI vs HC: *P*_adj_< 0.05, ATB vs LTBI: *P*_adj_> 0.05, ATB vs HC: *P*_adj_< 0.05), and these were categorized as EMMs. The levels of 151 out of 163 metabolites from area 1 (Fig. S2A) were changed in the ATB group compared with the HC and LTBI groups but did not differ significantly between the HC and LTBI groups (LTBI vs HC: *P*_adj_> 0.05, ATB vs LTBI: *P*_adj_< 0.05, ATB vs HC: *P*_adj_< 0.05), and these were categorized as LMMs. The levels of 21 metabolites from area 2 (Fig. S2A) were altered in one direction and differed significantly between each pair of groups (LTBI vs HC: *P*_adj_< 0.05, ATB vs LTBI: *P*_adj_< 0.05, ATB vs HC: *P*_adj_< 0.05), and these were categorized as PRMMs.

**Fig 2:**
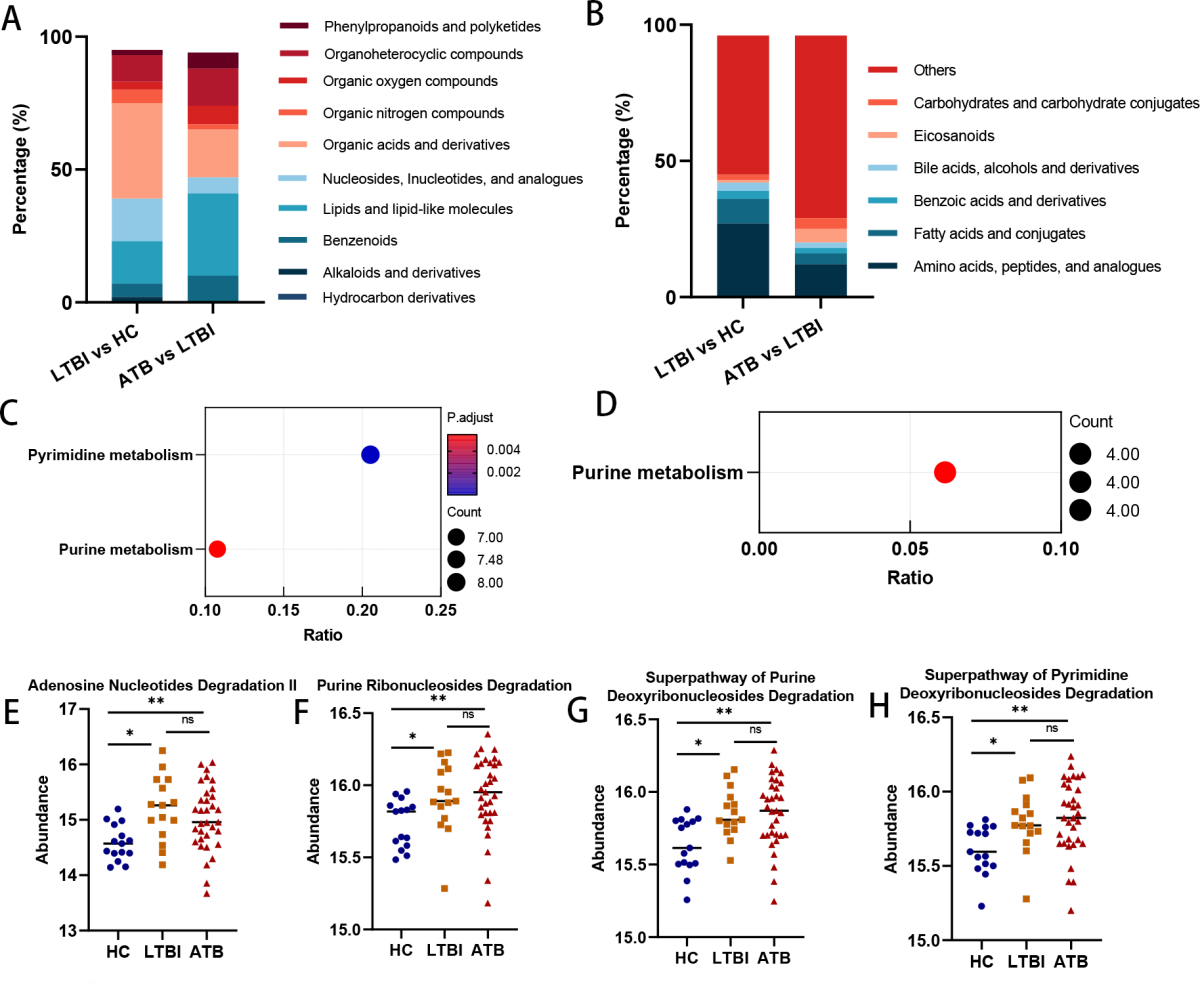
Compositional and functional alterations in gut metabolomic and microbiome profiling associated with TB progression. (**A**) The distributions of metabolites that were differentially abundant between the HC and LTBI groups and between the LTBI and ATB groups at the superclass level across categories. (**B**) The category distributions of differential metabolites at the subclass level. (**C**) Significant metabolic pathways for “EMMs + PRMMs” (metabolites differentially abundant between the HC and LTBI groups) based on KEGG functional analyses. The size of each bubble represents the number of metabolites enriched in the pathway, and the color gradient indicates the significance of enrichment. (**D**) Significant metabolic pathways for “PRMMs” based on KEGG functional analysis. (E–H) Bar plots comparing the levels of four nucleotide metabolism-related pathways calculated with PICRUSt2, including adenosine nucleotide degradation II, purine ribonucleoside degradation, superpathway of purine deoxyribonucleoside degradation, and superpathway of pyrimidine deoxyribonucleoside degradation, between the HC, LTBI, and ATB groups. Abbreviations: EMMs, early metabolic markers of LTBI; PRMMs, TB progression-related metabolic markers; KEGG, Kyoto Encyclopedia of Genes and Genomes.

Notably, we found that the metabolites in these three patterns exhibited pronounced depletion after TB infection, as 178 metabolites (78%; 54 EMMs, 105 LMMs, 19 PRMMs) were more abundant on average in the HC group. According to the classification criteria from the HMDB database, we noted that at the superclass level, “organic acids and derivatives” and “nucleosides, nucleotides, and analogues” were the categories to which metabolites that were differentially abundant between the LTBI and HC groups (EMMs + PRMMs) belonged (Fig. 2A), while at the subclass level, “amino acids, peptides, and analogues” accounted for 27% of the combined EMMs and PRMMs (Fig. 2B). With respect to metabolites with differences between the ATB and LTBI groups (LMMs + PRMMs), “lipids and lipid-like molecules” and “organic acids and derivatives” were prevalent at the superclass level (Fig. 2A). These findings highlight the complicated variations in fecal metabolic profiles that arise during the progression of TB.

In line with these metabolite profiles, three patterns of bacterial genera among the differentially abundant genera were classified as early bacterial markers of LTBI (EBMs), later bacterial markers of ATB (LBMs), and TB progression-related bacterial markers (PRBMs), which included 40, 31, and 17 genera, respectively (Table S2; Fig. S2B).

### Microbial functional analyses associated with TB

**Fig 3:**
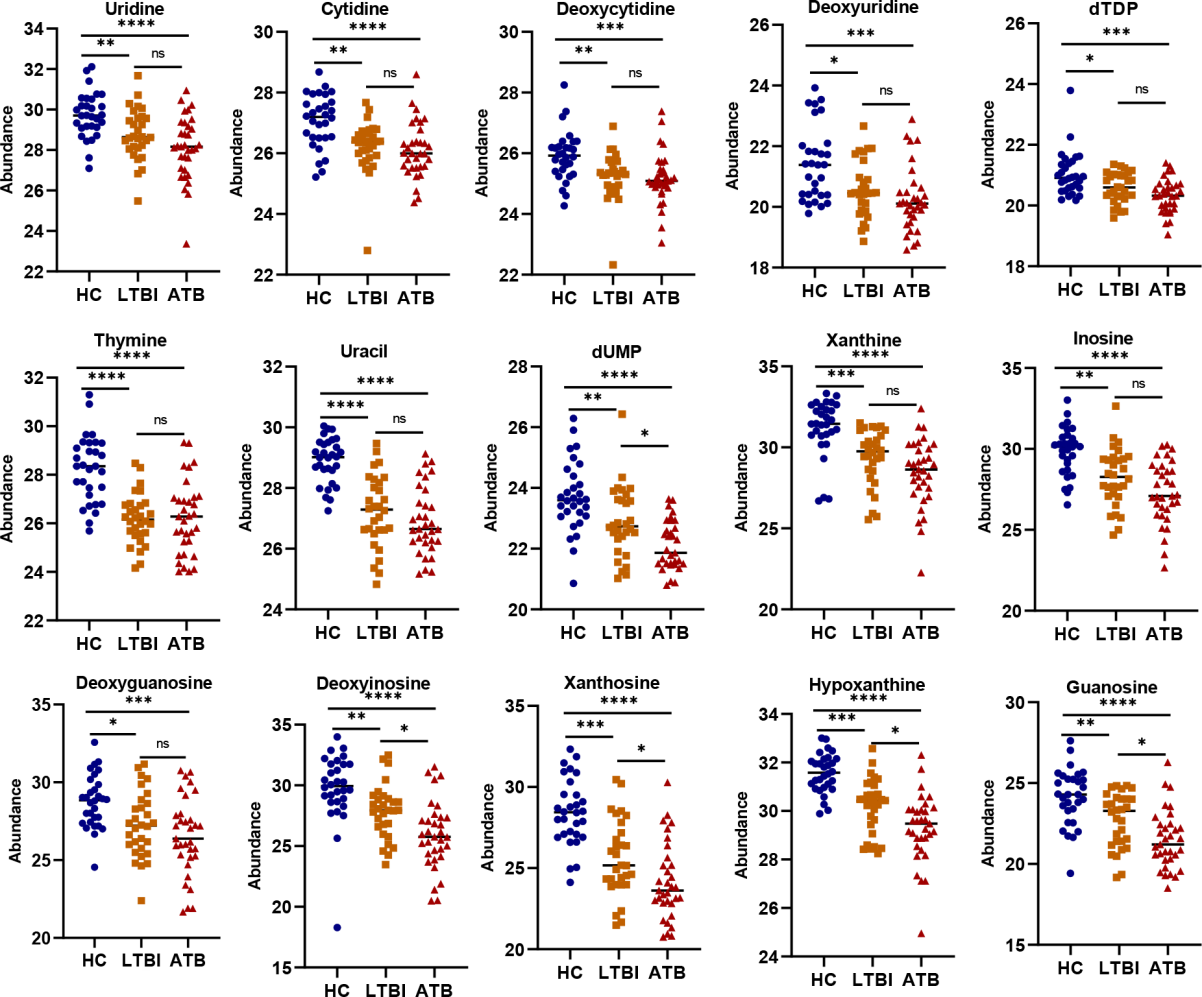
Levels of purine and pyrimidine metabolites in the three groups. Comparisons of fecal metabolites associated with purine and pyrimidine metabolism showing significant depletion after TB infection. Statistical analysis was performed using Kruskal–Wallis tests and Dunn’s *post hoc* test (^ns^*P >* 0.05, *P < 0.05, **P < 0.01, ***P < 0.001, ****P < 0.0001). Benjamini–Hochberg correction was employed to correct for multiple testing.

The three patterns of metabolites were subjected to KEGG enrichment analyses to investigate the associated metabolic pathways. For metabolites with differences between LTBI and HC (EMMs + PRMMs), pyrimidine and purine metabolism were significantly enriched (Fig. 2C; Table S3). However, for metabolites with differences between ATB and LTBI samples (LMMs + PRMMs), no metabolic pathway was enriched in a statistically significant manner (Table S4). When PRMMs were separately considered, only purine metabolism was enriched (Fig. 2D; Table S5). Among the metabolites associated with pyrimidine metabolism, uridine, cytidine, deoxycytidine, deoxyuridine, dTDP, thymine, and uracil were among the identified EMMs, whereas dUMP was among the identified PRMMs (Fig. 3; Table S1). Regarding the metabolites associated with purine metabolism, xanthine, inosine, and deoxyguanosine were EMMs, whereas deoxyinosine, xanthosine, hypoxanthine, and guanosine were PRMMs (Fig. 3; Table S1). In addition, it was found that the levels of fecal purine and pyrimidine metabolites decreased after TB infection, and it can, thus, be hypothesized that purines and pyrimidines may be degraded by gut microbes.

Interestingly, four nucleotide metabolism-related pathways, namely, adenosine nucleotide degradation II, purine ribonucleoside degradation, superpathway of purine deoxyribonucleoside degradation, and superpathway of pyrimidine deoxyribonucleoside degradation, were upregulated in the LTBI and ATB groups compared with the HC group. Moreover, these four pathways exhibited no significant difference between the LTBI and ATB groups (Fig. 2E through H; Table S6). These findings further suggested that the depletion of specific purine and pyrimidine metabolites is linked to the degradative activity of gut microbes in patients with LTBI and ATB.

### Interactions between disease-linked microbes and metabolites

Spearman’s correlation analyses were conducted to evaluate the relationships between differential genera (all microbial features in EBMs, LBMs, and PRBMs) and differential metabolites (all metabolic features in EMMs, LMMs, and PRMMs). A total of 20,064 associations were ultimately obtained and visualized (Tables S7 and S8; Fig. S2). Generally, these associations were in accordance with the relationship between the trends in metabolic and microbial content variability, and discordant associations, such as negative associations between metabolites and genera that both increased or decreased after TB infection, accounted for only 2% (407/20,064) of the total associations. Of the large number of possible associations between these metabolites and genera, only 60% were statistically significant (Benjamini–Hochberg correction, FDR threshold = 0.05). However, in the module of associations between PRBMs and PRMMs, 90% (322/357) were statistically significant, implying that the associations between these metabolites and genera are closely linked to the progression of TB. In this module of associations, *Incertae_Sedis* was both positively and strongly correlated with estrone, stercobilin, dodecanedioic acid, and tetradecanedioic acid (Spearman’s *r* > 0.7, *q* < 10^−7^).

**Fig 4:**
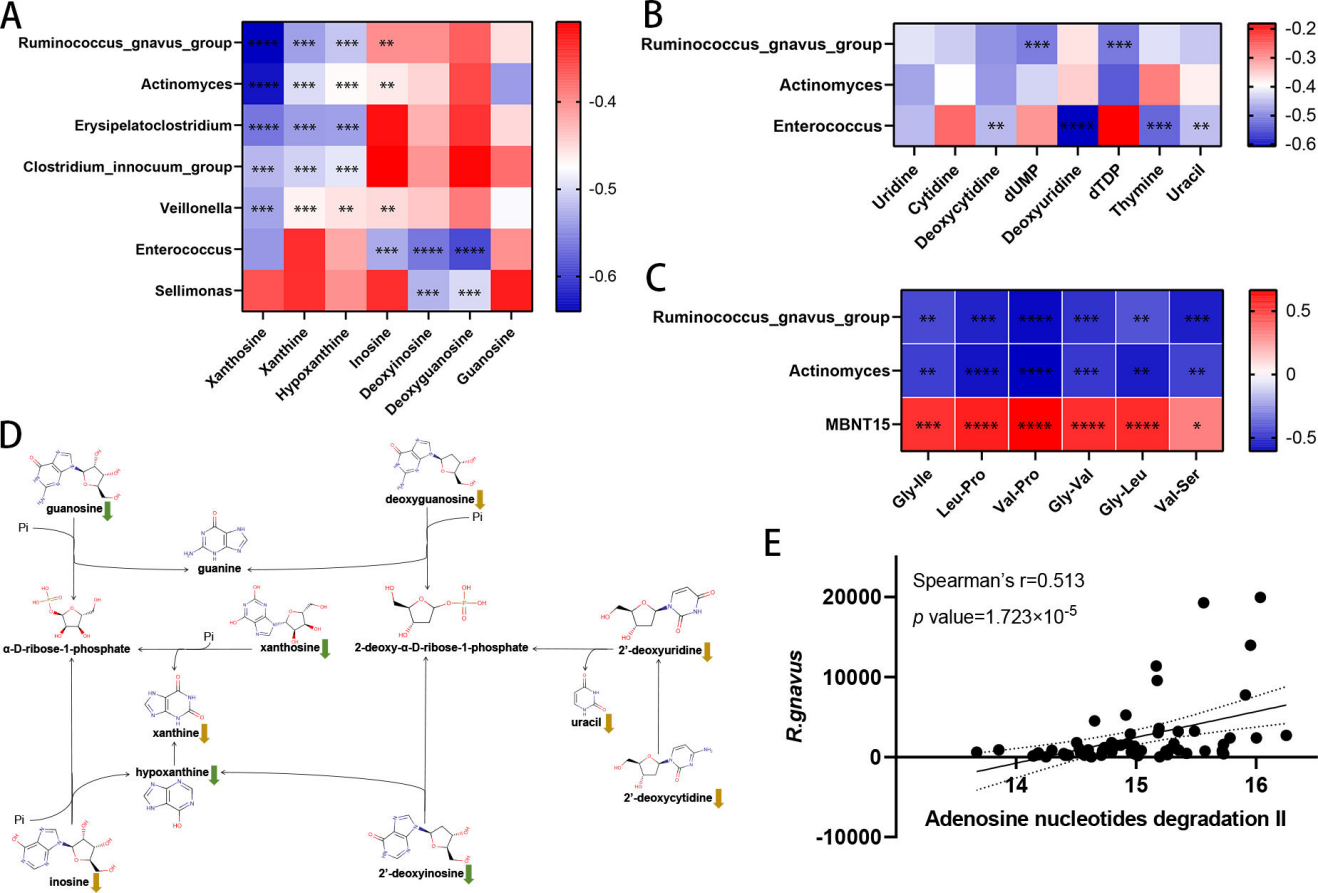
Correlations between gut microbial genera and metabolites. (**A**) Correlation analysis of differential genera and purine metabolites. Spearman’s correlation analyses were conducted to evaluate associations between genera and purine metabolites. The color gradient corresponds to the *r* value, where red represents the highest positive correlation and blue represents the lowest. (**B**) Correlation analysis of differential genera and pyrimidine metabolites. (**C**) Correlation analysis of differential genera and dipeptides. (**D**) Integrated metabolic pathways of purine and pyrimidine metabolism based on the MetaCyc database. (**E**) Spearman’s correlations between *Ruminococcus_gnavus_group* and “adenosine nucleotides degradation II.” Every dot represents a sample.

Next, we investigated which genera were strongly correlated with purine and pyrimidine metabolites. For purine metabolites, *Ruminococcus_gnavus_group, Actinomyces, Erysipelatoclostridium, Clostridium_innocuum_group,* and *Veillonella* were negatively correlated with xanthosine, xanthine, and hypoxanthine (Spearman’s |*r*| > 0.45, *q* < 10^−4^). *Ruminococcus_gnavus_group, Enterococcus*, *Actinomyces*, and *Veillonella* were negatively correlated with inosine (Spearman’s |*r*| > 0.40, *q* < 0.01). *Enterococcus* and *Sellimonas* were negatively correlated with deoxyinosine and deoxyguanosine (Spearman’s |*r*| > 0.5, *q* < 10^−4^). These findings indicate that these seven genera may be linked to purine depletion (Fig. 4A; Tables S7 and S8). With respect to pyrimidine metabolites (Fig. 4B; Tables S7 and S8), *Enterococcus* was negatively correlated with deoxycytidine, deoxyuridine, uracil, and thymine (Spearman’s |*r*| > 0.45, *q* < 0.01), while *Ruminococcus_gnavus_group* was negatively correlated with dUMP and dTDP (Spearman’s |*r*| > 0.5, *q* < 10^−4^).

Furthermore, certain genera were found to be associated with the dipeptides included among EMMs (Fig. 4C; Tables S7 and S8). Specifically, *MBNT15* was positively correlated with Gly-Ile, Leu-Pro, Gly-Leu, and Val-Pro (Spearman’s *r* > 0.5, *q* < 0.001), while *Actinomyces* was negatively correlated with Leu-Pro, Gly-Leu, and Val-Pro (Spearman’s |*r*| > 0.5, *q* < 10^−4^), and *Ruminococcus_gnavus_group* was negatively correlated with Val-Pro, Gly-Val, and Val-Ser (Spearman’s |*r*| > 0.5, *q* < 0.001).

Based on these findings, Spearman’s correlation, microbial PICRUSt2, and metabolic KEGG analyses were integrated to provide a holistic overview of purine and pyrimidine metabolism in the gut after TB infection. Notably, purine degradation and pyrimidine degradation were found to be linked (Fig. 4D). In the Spearman’s correlation analyses performed above, *Ruminococcus_gnavus_*group was found to be negatively correlated with the levels of xanthosine, inosine, hypoxanthine, and xanthine. Xanthine dehydrogenase accessory factor was also found in *Ruminococcus_gnavus_group* in PICRUSt2 analyses, suggesting that this genus may be associated with hypoxanthine degradation based on information from the MetaCyc database (Table S9). In addition, we observed a positive correlation between *Ruminococcus_gnavus_group* and the “adenosine nucleotides degradation II” pathway (Fig. 4E; Spearman’s |*r*| = 0.513, *q* = 1.723 × 10^−5^), which consists of the inosine-hypoxanthine-xanthine degradation axis. This finding further implies the close connection between *Ruminococcus_gnavus_group* and the depletion of inosine and hypoxanthine.

### Exploring metabolic biomarkers to discriminate between patients with LTBI and HCs

Our results demonstrated that the altered inosine-hypoxanthine-xanthine metabolic pathway in the gut emerged in the LTBI stage and was strongly associated with gut microbes. Therefore, inosine, hypoxanthine, and xanthine may serve as biomarkers for the early identification of LTBI.

**Fig 5:**
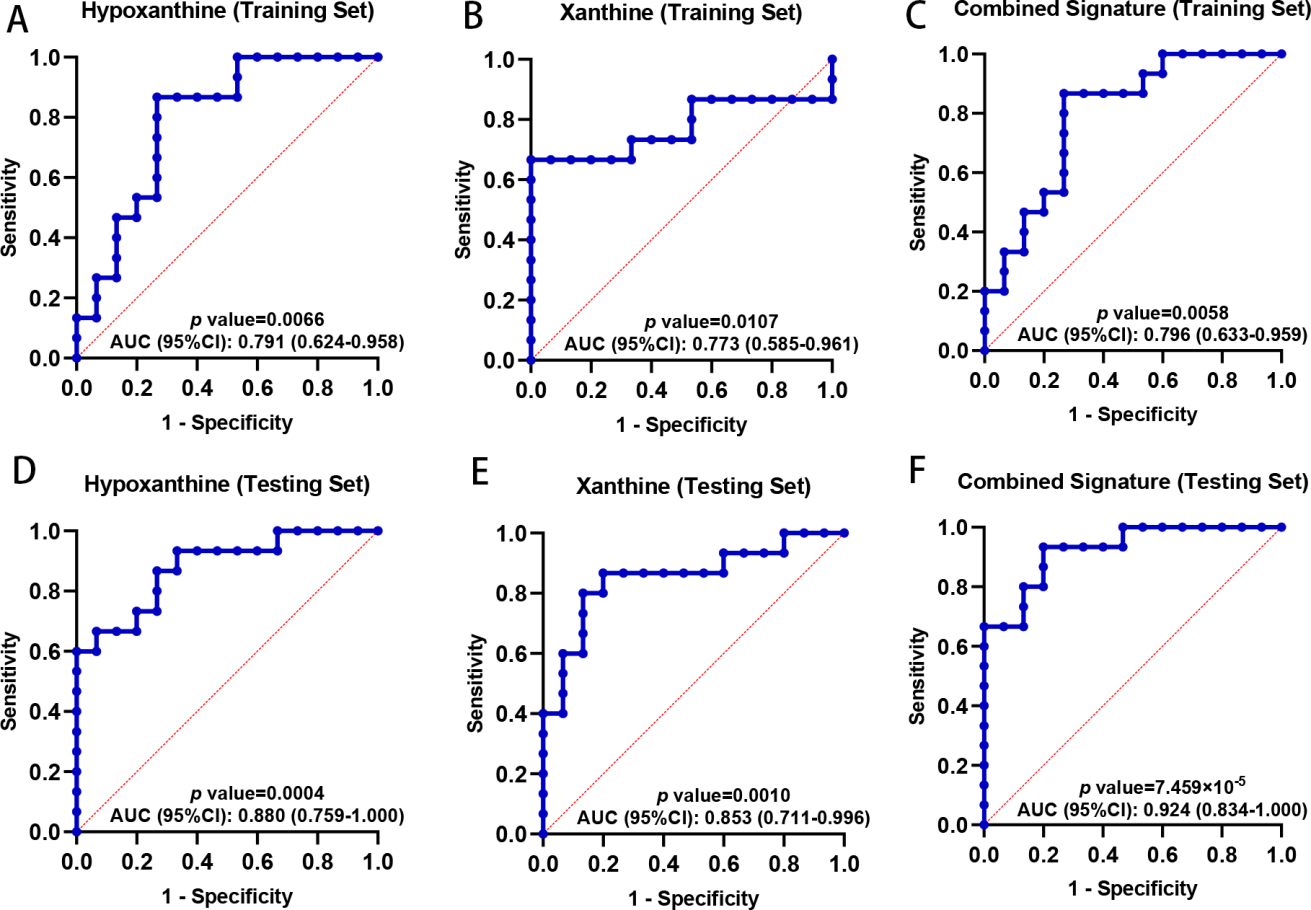
Diagnostic potential of fecal purine metabolites as a tool for discriminating between HCs and individuals with LTBI. (**A**) ROC curve for hypoxanthine in the training set, with an AUC of 0.791; (**B**) ROC curve for xanthine in the training set, with an AUC of 0.773; (**C**) ROC curve for the combined signature of hypoxanthine and xanthine in the training set, with an AUC of 0.796; (**D**) ROC curve for hypoxanthine in the testing set, with an AUC of 0.880; (**E**) ROC curve for xanthine in the testing set, with an AUC of 0.853; (**F**) ROC curve for the combined signature of hypoxanthine and xanthine in the testing set, with an AUC of 0.924. Abbreviations: ROC, receiver operating characteristic curve; AUC, area under the curve.

In both the training and testing sets, all three of these metabolites demonstrated excellent performance in distinguishing between HCs and patients with LTBI, and interestingly, in terms of the AUC value, these three metabolites always ranked, in descending order, as follows: hypoxanthine, xanthine, and inosine (Table S10). To further improve the discrimination efficacy of these biomarkers, LASSO regression was applied to construct a combined signature based on hypoxanthine and xanthine levels in the training set, yielding the following formula: score = (–0.123) × xanthine + (–0.773) × hypoxanthine*.* The AUC value for this combined signature was 0.796 in the training set, exceeding the diagnostic ability of hypoxanthine or xanthine alone (Fig. 5A through C). Then, the efficacy of this combined signature was tested in a testing set to investigate its reliability. Notably, the AUC value of the combined signature was 0.924, which was also greater than that of hypoxanthine or xanthine alone in the testing set (Fig. 5D through F). These results highlight the potential diagnostic value of the combined hypoxanthine and xanthine signature as a tool for identifying LTBI.

## DISCUSSION

Although numerous previous studies have described the compositional changes in gut microbes among patients with TB, a few have explored the functional shifts in the altered gut microbiota. Using high-throughput 16S rRNA gene amplicon sequencing and untargeted metabolomics data sets, we systematically summarized the microbial and metabolic changes in patients with LTBI and ATB compared with healthy individuals. Through multiple analyses, we found that purine and pyrimidine metabolism in the gut was altered as early as the LTBI stage, and this metabolic change was associated with gut dysbiosis. These observations provide new insights into the associations between gut dysbiosis and TB.

Alpha diversity can reflect the richness of taxa within a community. Previous studies have reported a decrease in alpha diversity in patients with ATB compared to healthy individuals. In a cohort of individuals with poorly controlled diabetes, the alpha diversity was similar between the LTBI and non-LTBI groups, but the beta diversity varied (24). Wang et al. investigated the difference in diversity during TB progression and found that the alpha diversity was not significantly different between the HC and LTBI groups, but the difference in beta diversity between these two groups was obvious (25). In our work, the results of the alpha and beta analyses were in line with previous findings, which suggested that the composition of gut microbiota shifts in the early stage of TB, which may provide new opportunities for TB prevention and therapy. A total of 10 phyla and 18 classes were apparently depleted at the LTBI stage, as evidenced by the compositional changes in gut microbes. With the exception of *Rhodothermia*, the other 18 classes branched from the 10 phyla. However, existing research on these classes is limited, and further exploration will be necessary to investigate the variations in these features.

The levels of 57 genera changed in the LTBI group compared to the HC group, among which 17 genera were found to be associated with TB progression as their levels also significantly changed from LTBI to ATB in the same trend as HC to LTBI. In accordance with the findings of previous studies, three of the 57 genera, including *Butyricimonas*, *Ruminococcus*, and *Roseburia*, were depleted in the LTBI and ATB groups when compared with the HC group (26, 27). These genera are probiotics and can produce SCFAs, a series of small molecules that can enhance respiratory immunity against pathogens. However, *Escherichia*, *Shigella,* and *Actinomyces,* which are opportunistic pathogens and disturb the homeostasis of mucosal barriers, were increased after TB infection(15). These findings suggest that severe dysbiosis of the gut microbiota has already emerged in the LTBI stage and may be associated with TB progression. Hence, regulating the disordered gut microbiota may represent a new strategy for the preventive treatment of LTBI.

In the current study, a total of 77 metabolites were differentially abundant between the HC and LTBI groups (EMMs + PRMMs), among which 22 belonged to the category “amino acids, peptides, and analogues” (2 EMMs, 20 PRMMs). Additionally, all of these amino acid metabolites were less abundant in the LTBI and ATB groups compared with the HC group. Numerous previous studies have explored the alterations in serum metabolic profiles in patients with ATB and have consistently observed a pattern of amino acid and peptide depletion (28–30). These findings based on serum metabolic alterations are in line with our results obtained through fecal untargeted metabolomics analyses, indicating that there are consistent alterations in amino acids in both plasma and feces among patients with TB relative to HCs and that this trend can be observed as early as the LTBI stage. Nutritional deficiency has been recognized as an important factor contributing to TB, as undernutrition has been broadly observed among patients with TB in developing countries with high burdens of disease (31–34). Undernutrition, especially the lack of protein supplementation, can weaken the host immune response to *M. tb* and, thus, may contribute to the progression of TB. In animal models with insufficient protein supplementation, lung immunity against *M. tb* has been shown to be impaired by multiple mechanisms compared to control groups provided with sufficient protein, including reduced activation of Th1 responses, impaired macrophage phagocytosis, decreased dendritic cells, impaired antigen presentation, and disorganized granulomas (34). In the current study, various dipeptides were found to be less abundant in the LTBI and ATB groups, especially those composed of branched-chain amino acids (BCAAs), which play an important role in protein synthesis (35). Furthermore, the level of dipeptides was significantly associated with several gut genera. Given that the nutritional status was comparable among the HC, LTBI, and ATB groups, we inferred that the decrease in these amino acids in infected individuals was associated with gut microbes. Recently, the role of the gut microbiota in amino acid metabolism has been extensively explored in healthy individuals and those with many diseases, confirming the close interactions between many amino acids, including tryptophan and BCAAs, and gut microbes (36–39). However, a few studies have systematically researched the associations between altered amino acid metabolism and gut microbes in TB. Therefore, our findings provide new insight and an entry point for further studies regarding the changes in amino acid catabolism among gut microbes in patients with TB.

PICRUSt2 analysis revealed the active degradation of purine and pyrimidine metabolites among gut microbes in patients with LTBI and ATB. Previous studies have revealed that purine and pyrimidine metabolites play important roles in immune modulation. Inosine, produced by *Bifidobacterium pseudolongum* in the gut, has been shown to have antimicrobial ability against *nontuberculous mycobacterial* (NTM) infections, and oral intake of inosine can augment the antimicrobial immune response against NTM in macrophages (40). Inosine can also enhance IL-1β secretion by macrophages and drive inflammatory activation (41). This pro-inflammatory process may be vital for eliminating *M. tb,* as macrophages are a primary mediator of *M. tb* clearance. In another study, gut-derived inosine was shown to bind to the A_2A_ receptor on T cells and trigger the A2AR-cAMP-PKA signaling cascade, thus promoting Th1 differentiation and increasing the efficacy of immune checkpoint blockade therapy against tumors (42). Inosine was also found to inhibit UBA6 on tumor cells, thus increasing tumor immunogenicity and making tumor cells more prone to T cell killing (43). These results indicate that inosine can augment host immunity against antigens. In our study, the levels of inosine were found to be decreased in patients with LTBI and ATB. Previous findings have suggested that a decrease in inosine weakens the immune response of T cells and macrophages against *M. tb*, which we posit may be associated with *M. tb* susceptibility. However, further experimental exploration is needed to verify the immune function of inosine against *M. tb*. Pyrimidine metabolites were also found to be closely associated with host immune function. Yulia Kushnareva et al. found that the depletion of the *ISOC1* in Th1 cells perturbs intracellular pyrimidine metabolism and further reduces the secretion of IFN-γ and IL-17 (44). Moreover, when these defective Th1 cells were supplied with exogenous cytidine or uridine, the production of IFN-γ and IL-17 increased. According to previous research, individuals with IFN-γ deficiency are prone to mycobacterial diseases (44, 45). IFN-γ-producing Th1 cells have been shown to be essential in *M. tb* control and account for more than 50% of *M. tb*-specific memory T cells (44, 46). These experimental findings highlight the important role of pyrimidine metabolites in the immune function of Th1 cells, while a sufficient pyrimidine supply is crucial to enhance the immune response against *M. tb*. Additionally, our study found that uracil levels in the gut have already decreased by the LTBI stage. In the research conducted by Kyung-Ah Lee et al., gut microbe-derived uracil was shown to activate innate immunity in the gut, which is involved in maintaining microenvironmental homeostasis and eliminating pathogenic bacteria (47). As increasing evidence suggests that patients with lung diseases are more likely to simultaneously suffer from intestinal dysfunction (13, 48), the variations in uracil content may mediate this pathological process. Despite the fact that none of the individuals in our cohort had gastrointestinal diseases, the level of uracil is a promising early biomarker of gut dysfunction and related diseases in individuals with *M. tb* infection. However, further studies will be needed to verify the immune function and clinical value of these pyrimidine metabolites.

Compared with the HC group, higher levels of *Ruminococcus_gnavus_group* (*R. gnavus*) were observed in the LTBI and ATB groups, meaning that the level of gut *R. gnavus* was altered in the early stages of TB despite a lack of any apparent symptoms. In the current study, *R. gnavus* abundance was negatively correlated with inosine, xanthosine, xanthine, and hypoxanthine levels. Notably, *R. gnavus* was positively associated with the “adenosine nucleotides degradation II” pathway, which is the inosine degradation pathway, further emphasizing the tight association between *R. gnavus* and purine metabolism. In a study focusing on gut microbial and metabolic shifts in patients with chronic kidney disease and hyperuricemia, *R. gnavus* was found to be negatively correlated with xanthosine, xanthine, and deoxyinosine levels (49). These relationships between *R. gnavus* and purine metabolites are in line with our results, further suggesting that *R. gnavus* is crucial for purine degradation in the gut. Recently, the level of *R. gnavus* in the gut was found to vary with many diseases, including gastrointestinal diseases, inflammatory skin diseases, neurological disorders, and other respiratory diseases in addition to TB (50). For example, higher levels of *R. gnavus* have been observed in patients infected with SARS-CoV-2 and those with long-term complications (51, 52). In a study conducted by Chua et al., elevated levels of *R. gnavus* were associated with asthma and were observed before the onset of allergic symptoms. In mice intragastrically transplanted with *R.gnavus*, the intensive accumulation of Th2 cells and related cytokines in lung tissues was observed. Further experiments revealed that colonic *R. gnavus* primarily stimulates the epithelium to secrete cytokines, thereby promoting the differentiation of Th2 cells and secretion of Th2-related cytokines, which consequently circulate to the lung to mediate inflammation (53). However, Th2 responses cannot restrain the growth of *M.tb,* and this can lead to ATB becoming a persistent chronic condition (54, 55). Thus, gut *R.gnavus* may impair the immune response against *M.tb* by inducing the production of Th2 cells, thereby maintaining the LTBI state. These results demonstrate that gut *R. gnavus* is strongly associated with respiratory inflammation and may influence immune responses. Importantly, Hu et al. found that the level of gut *R. gnavus* in patients with TB decreased after the administration of anti-TB drugs (56). Based on this prior evidence, interventions targeting gut *R. gnavus* and associated purine degradation may represent a viable new anti-TB strategy.

However, there are some limitations to our study. First, this study explored the associations between gut microbes and metabolites without investigating the causal relationships associated therewith. Second, although we analyzed the correlations between microbes and metabolites from multiple perspectives, our sample size was small, and no further validation was performed due to funding limitations. Third, all of the individuals in this study were from the same city, so a multicenter study is needed to verify our findings in the future. Fourth, shotgun metagenomics sequencing was not performed to profile the microbiome, and such data are more suitable for predicting the function of gut microbes. Although the results of microbial functional analysis are consistent with the metabolomics findings, using metagenomics data for functional prediction is more robust. Additionally, the microbial composition of lung or bronchoalveolar lavage fluid samples was not profiled, which is essential for accurate data interpretation. Finally, we did not conduct animal experiments to investigate how intestinal *R. gnavus*, purine metabolism, and pyrimidine metabolism influence immune responses against *M. tb* in the lung. Despite these limitations, our findings still expand our understanding of how the gut microbiota and associated metabolites change and interact with each other in patients with LTBI and ATB. This study also provides new insights and many actionable hypotheses regarding the role of specific metabolites and their potential microbial partners in the pathogenesis, diagnosis, and treatment of TB.

### Conclusion

In the present study, we systematically characterized the gut microbial and metabolic differences between healthy individuals, those with LTBI, and patients with ATB. These alterations in microbiome and metabolomic profiles were closely correlated during the progression of TB. We also found that purine and pyrimidine metabolism was perturbed as early as the LTBI stage. The degradation of purine and pyrimidine metabolites, especially the inosine-hypoxanthine-xanthine degradation pathway, was significantly associated with gut microbes represented by *R. gnavus*. Notably, the metabolic signature composed of hypoxanthine and xanthine exhibited excellent performance in discriminating between the HC and LTBI groups, showing great clinical value in the early, rapid, and noninvasive diagnosis of TB. Our findings provide novel strategies for the diagnosis and management of LTBI. However, further experimental and cohort studies are still needed to explore the feasibility of such strategies and the deeper mechanistic basis for our findings.

## Data Availability

The raw sequence data reported in this paper have been deposited in the Genome Sequence Archive of the National Genomics Data Center, China National Center for Bioinformation/Beijing Institute of Genomics, and Chinese Academy of Sciences (GSA-Human: CRA015625) and are publicly accessible at https://ngdc.cncb.ac.cn/gsa/search?searchTerm=CRA015625. Metabolomic profiling is shown in Table S11.

## SUPPLEMENTAL MATERIAL

Fig. S1 msystems.00812-24-s0001.pdf Heatmap of microbial features at the phylum and class levels.

Fig. S2 msystems.00812-24-s0002.pdf Screening of differential microbial genera and metabolites.

Fig. S3 msystems.00812-24-s0003.pdf Correlation analysis of differential genera (all microbial features in EBMs, LBMs, and PRBMs) and differential metabolites (all metabolic features in EMMs, LMMs, and PRMMs).

Supplemental Tables msystems.00812-24-s0004.xlsx Tables S1-S11.

ASM does not own the copyrights to Supplemental Material that may be linked to, or accessed through, an article. The authors have granted ASM a non-exclusive, world-wide license to publish the Supplemental Material files. Please contact the corresponding author directly for reuse.
