# Supplementary material for: Alterations in purine and pyrimidine metabolism associated with latent tuberculosis infection: insights from gut microbiome and metabolomics analyses
Source: mSystems. 2024 Oct 22;9(11):e00812-24. doi: 10.1128/msystems.00812-24 (PMC11575419; doi:10.1128/msystems.00812-24)

Additional file 2: Additional Fig. 1. Heatmap of microbial features at the phylum and class levels. (A) Heatmap showing the microbial features depleted to almost zero in the LTBI stage (framed with red lines) at the phylum level. **(B)** Heatmap showing the microbial features depleted to almost zero in the LTBI stage at the class level.

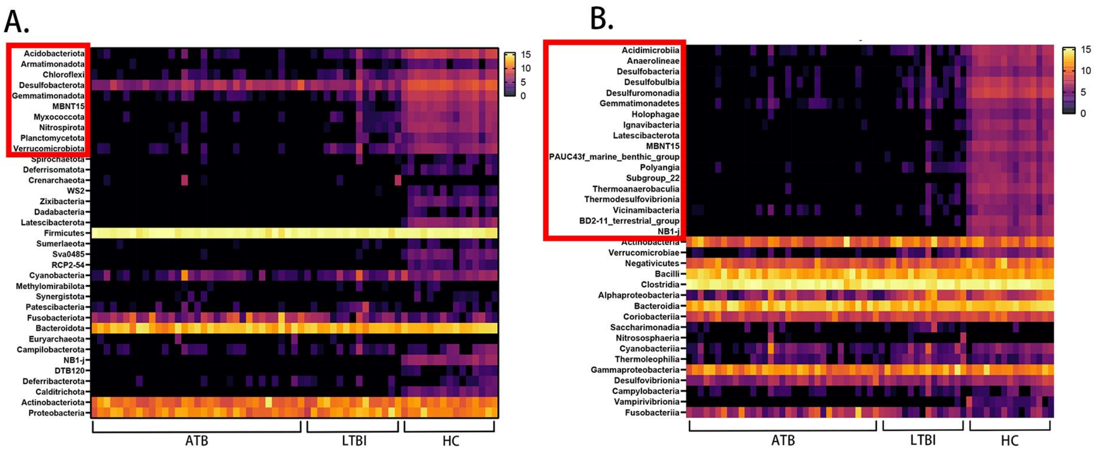

Supplement: Fig. S1 — Heatmap of microbial features at the phylum and class levels. [file msystems.00812-24-s0001.pdf]
