# Supplementary material for: Alterations in purine and pyrimidine metabolism associated with latent tuberculosis infection: insights from gut microbiome and metabolomics analyses
Source: mSystems. 2024 Oct 22;9(11):e00812-24. doi: 10.1128/msystems.00812-24 (PMC11575419; doi:10.1128/msystems.00812-24)

Additional file 3: Additional Fig. 2. Screening of Differential microbial genera and metabolites. (A) Venn plot showing differential genera between every two groups; **(B)** Venn plot showing differential metabolites between every two groups. The barplots attached to venn plots visualized the level differences of three patterns of genera (EBMs, LBMs and PRBMs) or metabolites (EMMs, LMMs and PRMMs) between HC, LTBI and ATB. Abbreviations: EBMs, early bacterial markers of LTBI; LBMs, later bacterial markers of ATB; PRBMs, TB progression-related bacterial markers; EMMs, early metabolic markers of LTBI; LMMs, later metabolic markers of ATB; PRMMs, TB progression-related metabolic markers; HC, healthy controls; LTBI, latent tuberculosis infection; ATB, active tuberculosis

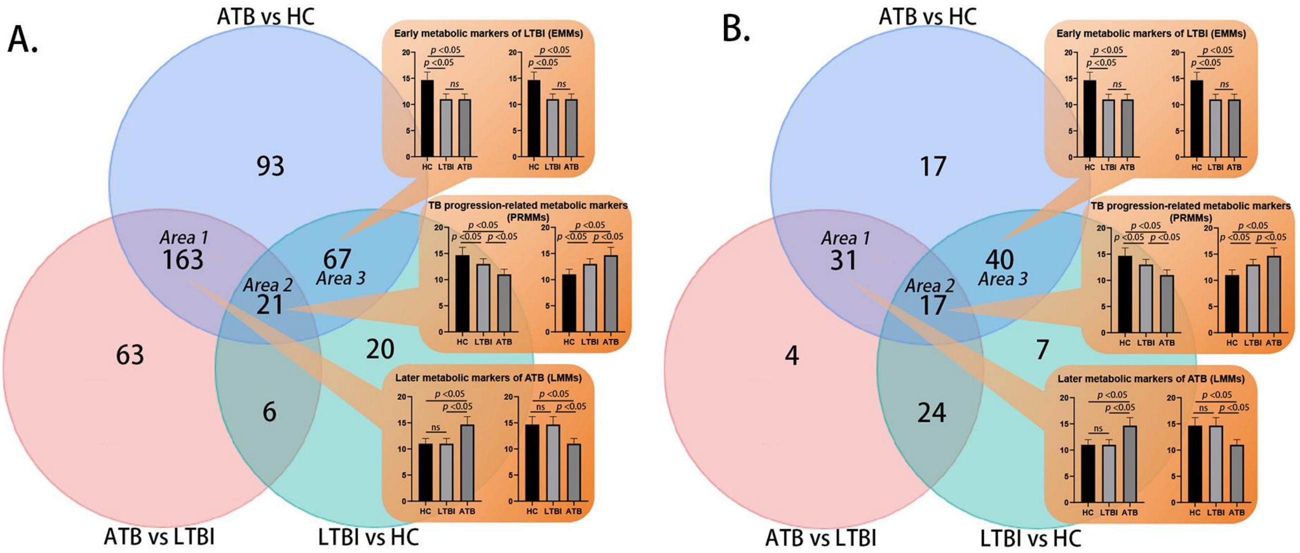

Supplement: Fig. S2 — Screening of differential microbial genera and metabolites. [file msystems.00812-24-s0002.pdf]
