# Supplementary material for: Alterations in purine and pyrimidine metabolism associated with latent tuberculosis infection: insights from gut microbiome and metabolomics analyses
Source: mSystems. 2024 Oct 22;9(11):e00812-24. doi: 10.1128/msystems.00812-24 (PMC11575419; doi:10.1128/msystems.00812-24)

Additional file 4: Additional Fig. 3. Correlation analysis of differential genera (all microbial features in EBMs, LBMs, and PRBMs) and differential metabolites (all metabolic features in EMMs, LMMs, and PRMMs). Spearman's correlation analysis was performed to evaluate associations. The color gradient corresponds to the r value, with red and blue respectively representing the strongest positive correlation and the weakest. Abbreviations: EBMs, early bacterial markers of LTBI; LBMs, later bacterial markers of ATB; PRBMs, TB progression-related bacterial markers; EMMs, early metabolic markers of LTBI; LMMs, later metabolic markers of ATB; PRMMs, TB progression-related metabolic markers

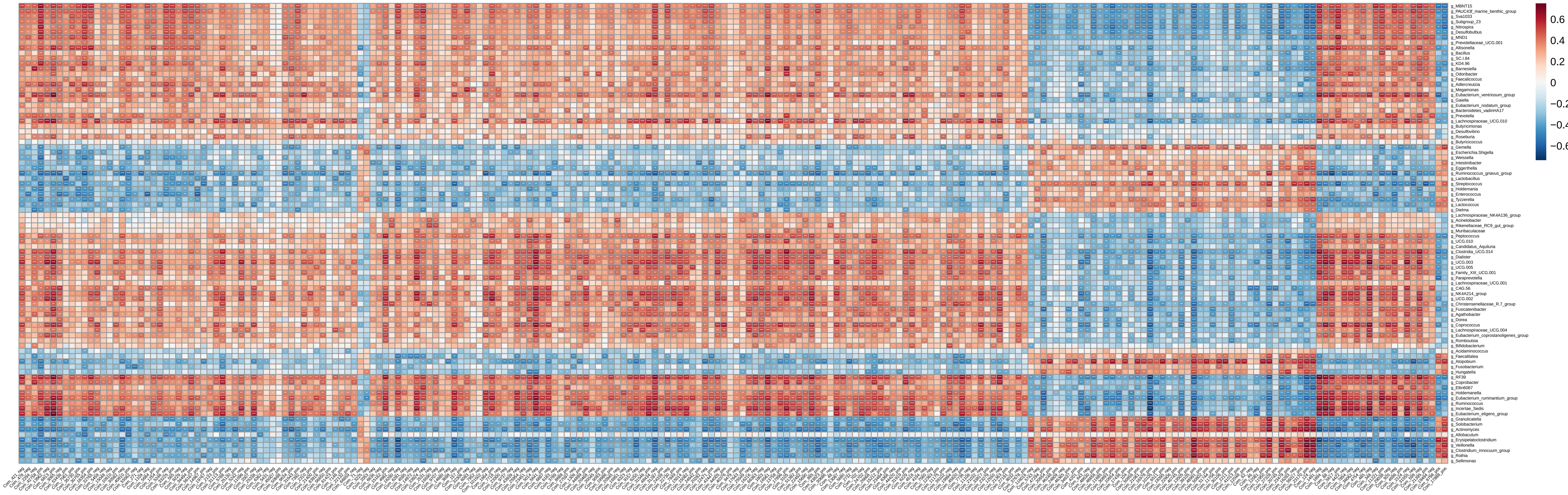

Supplement: Fig. S3 — Correlation analysis of differential genera (all microbial features in EBMs, LBMs, and PRBMs) and differential metabolites (all metabolic features in EMMs, LMMs, and PRMMs). [file msystems.00812-24-s0003.pdf]
